# Methane: Fuel or Exhaust at the Emergence of Life?

**DOI:** 10.1089/ast.2016.1599

**Published:** 2017-10-01

**Authors:** Michael J. Russell, Wolfgang Nitschke

**Affiliations:** ^1^ Jet Propulsion Laboratory, California Institute of Technology, Pasadena, California.; ^2^ CNRS/Aix-Marseille University, BIP UMR 7281, IMM FR 3479, Marseille, France.

## Abstract

As many of the methanogens first encountered at hydrothermal vents were thermophilic to hyperthermophilic and comprised one of the lower roots of the evolutionary tree, it has been assumed that methanogenesis was one of the earliest, if not *the* earliest, pathway to life. It being well known that hydrothermal springs associated with serpentinization also bore abiotic methane, it had been further assumed that emergent biochemistry merely adopted and quickened this supposed serpentinization reaction. Yet, recent hydrothermal experiments simulating serpentinization have failed to generate methane so far, thus casting doubt on this assumption. The idea that the inverse view is worthy of debate, that is, that methanotrophy was the earlier, is stymied by the “fact” that methanotrophy itself has been termed “reverse methanogenesis,” so allotting the methanogens the founding pedigree. Thus, attempting to suggest instead that methanogenesis might be termed reverse methanotrophy would require “unlearning”—a challenge to the subconscious! Here we re-examine the “impossibility” of methanotrophy predating methanogenesis as in what we have termed the “denitrifying methanotrophic acetogenic pathway.” Advantages offered by such thinking are that methane would not only be a fuel but also a ready source of reduced carbon to combine with formate or carbon monoxide—available in hydrothermal fluids—to generate acetate, a target molecule of the first autotrophs. And the nitrate/nitrite required for the putative oxidation of methane with activated NO would also be a ready source of fixed nitrogen for amination reactions. Theoretical conditions for such a putative pathway would be met in a hydrothermal green rust-bearing exhalative pile and associated chimneys subject to proton and electron counter gradients. This hypothesis could be put to test in a high-pressure hydrothermal reaction chamber in which a cool carbonate/nitrate/nitrite-bearing early acidulous ocean simulant is juxtaposed across a precipitate membrane to an alkaline solution of hydrogen and methane. Key Words: Green rust—Methanotrophy—Nitrate reduction—Emergence of life. Astrobiology 17, 1053–1066.

*A likely impossibility is always preferable to an unconvincing possibility*.

—Aristotle

## 1. Introduction

Motivated by growing concern as to whether methane was the fuel or the waste of emergent life, the alkaline vent theory (AVT) has evolved through a number of changes to its present, yet still tentative, formulation (Nitschke and Russell, 2013). As first envisioned, the AVT carried the expectation that serpentinization would provide the fuels hydrogen and methane as usable electron donors, as well as other small molecules, in a low-entropy hydrothermal alkaline feed through nonmagmatic submarine springs to spontaneously precipitated porous mineral mounds.

Under this early view, pores in these mounds were the “culture chambers” in which life emerged (Russell *et al.*, 1989, 1994). It had been further ventured, following Degens, that hydrothermal minerals would have “catalyzed” the synthesis of “peptides, polysaccharides, lipids, and nucleic acids, which would have been discharged to the hydrosphere” (Degens, 1979; Russell *et al.*, 1989). This view was elaborated as “metabolism quickens, by many orders of magnitude, oxidation, and reduction reactions on our planet”… (and)… “links can be assumed between the rather slow, low-temperature reactions of geochemistry and the quickened reactions of early biochemistry” (Russell *et al.*, 2003).

Following on from this supposition, Martin and Russell (2007) mooted the idea that microbial methanogenesis—assumed then to have been the most ancient form of archaeal metabolism—had emerged as a “quickening” of the abiotic serpentinization reaction presumed to occur at moderate hydrothermal temperatures. That methanogens occupied one root of the evolutionary tree and appeared to be thermophilic, or even hyperthermophilic, was considered to support the notion (Amend and Shock, 2001; Ciccarelli *et al.*, 2006).

Yet, a nagging and anxious thought regarding the plausibility of these later ideas was: Why would methane be generated in competition with the high concentration of the same volatile in the alkaline effluent? That is, why would methane synthesis be driven against that concentration gradient? In addition, concerns arising from multiple sources—kinetic considerations, isotopic investigations, possible contaminations, biotic sources, and false positives in experiments, as well as phylogenetic analyses—have thrown doubt on the assumption that methanogenesis was foundational to the archael domain (Woese *et al.*, 1990; Kelley and Früh-Green, 1999; Seewald *et al.*, 2006; McCollom and Seewald, 2007; Proskurowski *et al.*, 2008; Shock and Canovas, 2010; Lazar *et al.*, 2012; Paukert *et al.*, 2012; Nitschke and Russell, 2013; Reeves *et al.*, 2014; Suda *et al.*, 2014; McDermott *et al.*, 2015; Seyfried *et al.*, 2015; McCollom and Donaldson, 2016). Most arresting was the fact that Proskurowski *et al.* (2008) demonstrated an absence of radiocarbon in methane in the Lost City fluids, strongly implying that this methane is not derived from CO_2_ delivered by percolated and convecting ocean waters, but that any hydrocarbon source of the methane, or the methane itself, was intrinsic to, or had lodged within, the ocean crust and merely been released to, but not generated by, the circulating fluids.

That methane is a major volatile, second only to hydrogen in terrestrial alkaline springs, was already well known when the AVT was first formulated (Moiseyev, 1968; Neal and Stanger, 1984). Convecting, advecting, and/or artesian aqueous fluids fed from surface waters and exhaling from serpentinizing terrestrial ultramafic rocks are always alkaline and always enriched in hydrogen (≤25 m*M*) and methane (≤3 m*M*) (Abrajano *et al.*, 1988; Etiope *et al.*, 2012; Greenberger *et al.*, 2015; Konn *et al.*, 2015; Seyfried *et al.*, 2015).

There is sturdy contextual and experimental evidence to suppose that the hydrogen was, and is, generated still through the exergonic oxidation of iron in olivine and, to a lesser degree, in the more recalcitrant orthopyroxene, by water (Coveney *et al.*, 1987; McCollom and Bach, 2009), with recent research possibly adding iron-rich spinels to the mixture (Mayhew *et al.*, 2013). It has also been argued that a proportion of the hydrogen so released goes on to reduce carbonate or bicarbonate, originally present in the same circulating fluid, to methane in a mechanism akin to the Sabatier reaction (Horita and Berndt, 1999; Neubeck *et al.*, 2011; Wang and Gong, 2011; Etiope *et al.*, 2012). Indeed, multiple investigations to determine whether methane could be generated through the reduction of CO_2_ species have been made and several showed that CO, CO_2_, or HCO_3_^−^ species can be reduced by iron–nickel alloys to form methane under low-temperature (<200°C) alkaline hydrothermal conditions (Horita and Berndt, 1999; Wang and Gong, 2011). Certainly, reduction of such precursors to methane, while facing substantial kinetic obstacles, is thermodynamically favorable (Shock, 1992; Maden, 2000; Lyons *et al.*, 2005; McCollom and Seewald, 2007).

Moreover, we were aware from the calculations of Shock (1992) that serpentinization reactions taking place in off-ridge systems at moderate temperatures of 120–150° are more favorable to the thermodynamic drive to CO_2_ reduction than those at higher temperature—indeed, the lower the better in terms of the Gibbs free energy though not in terms of kinetics (cf., Herschy *et al.*, 2014).

Yet, the recent article by McCollom and Donaldson (2016) has thrown what was a seemingly straightforward segue from geochemistry to biochemistry in some disarray. In experiments lasting >20 weeks, they failed to observe the generation of methane from CO_2_, thus placing the idea of methanogenesis as “an observable homologue at hydrothermal vents,” and thereby its foundational status, in doubt (*pace* Sousa and Martin, 2014). Compounding the uncertainties regarding the original AVT formulation is the suggestion of Windman *et al.* (2007) that hydrothermal formate, rather than hydrogen, was an initial fuel. Being thus forced to rethink our assumptions led us to attempt to make more sense of the stepped transitions from mineral to life–life that still extracts its inorganic components ultimately from mineral, while still being driven by redox disequilibria (Leduc, 1911; Nitschke and Russell, 2009, 2013; cf., McGlynn, 2017).

## 2. So What Were the First Fuels and Oxidants?

The original idea that life emerged autogenically into the very first autotrophs has a long pedigree (Traube, 1867; Pfeffer, 1877; Darwin, C, in Darwin, F, 1888; Haeckel, 1892; Mereschkowsky, 1910; Leduc, 1911; Goldschmidt, 1952)—a view beclouded for some 80 years by Oparin's and Haldane's primordial soup (Lane *et al.*, 2010). Autotrophic views resurfaced in the context of submarine hydrothermal vents late last century with, for example, gradients across hydrothermal chimneys inducing the anaerobic utilization of H_2_ and native sulfur as well as the production and consumption of CH_4_ (Corliss *et al.*, 1981; Baross and Hoffman, 1985). In a similar vein, Wächtershäuser (1988) called upon a putative pyrite reaction to supply electrons for CO_2_ fixation. In contrast, Russell *et al.* (1989) appealed directly to hydrothermally generated organic molecules as well as to hydrogen and methane as fuels.

However, since McCollom and Donaldson (2016) concluded that the reduction of CO_2_ all the way to CH_4_ during serpentinization may take thousands of years, we reconsider previous researches that assumed hydrothermal methane to have been variously (i) leached, or cracked, from primary abiotic and/or biotic organic precursors within the ultramafic crustal rocks, (ii) reduced from mantle-derived CO_2_ in the lower-to-mid crust and trapped thereafter in fluid inclusions, or (iii) even leached from under-thrust sediments and metasediments or Precambrian continental lithosphere. All these studies force a reassessment of what might have been the very first steps to life (Watanabe *et al.*, 1983; Pineau and Mathez, 1990; Proskurowski *et al.*, 2008; McCollom, 2013; Lollar *et al.*, 2014; Suda *et al.*, 2014).

As formate is the only rapidly produced organic molecule through CO_2_ reduction in conditions at, or simulating those at, an alkaline vent (Lang *et al.*, 2010, 2012; Seyfried *et al.*, 2015), and because McCollom and Donaldson (2016) also threw doubt on the assumption that hydrogen is produced rapidly through the serpentinization reaction, we no longer presuppose an immediate quickening or takeover of the hydrogenase reaction either! Perhaps the biological hydrogenation only came into play after the first steps of metabolism were mounted (Schuchmann and Muller, 2013). Otherwise, following Windman and coworkers' suggestion (Windman *et al.*, 2007), formate could be considered as both a fuel *and* as another source of carbon.

**FIG. 1.**
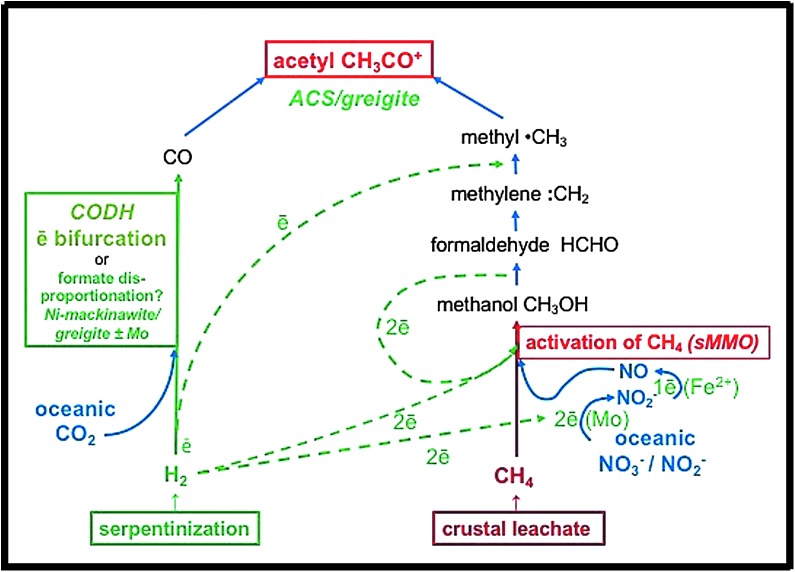
Simplified reaction steps of the putative denitrifying methanotrophic acetogenesis model based on Nitschke and Russell (2013, figures 3–5). Methane is produced by hydrothermal leaching of cracked carbon material previously residing in the crust. Hydrogen and formate are generated through the reduction of water during serpentinization. Hydrogen provides electrons for the reduction of HCO_3_^−^ to HCOO^−^. Formate disproportionates to CO and water as pH drops (Keene, 1993). The reduction of nitrate to NO (Kampschreur *et al.*, 2011) drives the putative oxidation of methane to methanol and the hydrogenation of methylene to a methyl group. The methyl group reacts with the CO to produce activated acetate (Chistoserdova *et al.*, 2009). Although this denitrifying methanotrophic (right hand) path to acetate looks complicated, the high electron mobilities (tunneling and bifurcations) within semiconducting GR allow improvization toward the best, if intricate, pathways and thereby long-range charge transport (Marcus, 1964; Wander *et al.*, 2007; Nitschke and Russell, 2009, 2011, 2013; Ruby *et al.*, 2010; Génin *et al.*, 2012). Water and heat are among the waste products. The active sulfidic centers of metalloenzymes CODH (carbon dioxide dehydrogenase) and ACS (acetyl coenzyme synthase) are affine with the structure of nickeliferous greigite (Russell and Hall, 2006; Cao *et al.*, 2009; Nitschke and Russell, 2013; Bassegoda *et al.*, 2014; Roldan *et al.*, 2015), whereas sMMO (soluble methane monooxygenase) is a di-iron enzyme with similarities to the structure of the more reduced form of GR, fougerite (Fe^II^_4_Fe^III^_2_(OH)_12_CO_3_•3H_2_O) (Nitschke *et al.* 2013). GR, green rust.

Although these findings threaten the simple “rocky-roots,” or purely reductive form of the acetogenic pathway (Russell and Martin, 2004; Sousa and Martin, 2014), were CO_2_ to be reduced to only formate during serpentinization, then we are left with the tributary to the methyl group being derived oxidatively from CH_4_, although still arriving at the target molecule of the acetyl coenzyme-A pathway, acetate, through methylation of CO (Nitschke and Russell, 2013; Sojo *et al.*, 2015). Thus, in our opinion, abandonment of the methanogenic aspect of the acetyl-coA pathway as the initiator of metabolism leaves only one alternative, namely, a denitrifying methanotrophic acetogenic pathway (DMAP) (Nitschke and Russell, 2013) (Fig. 1). Under this view, hydrothermal methane is considered a fuel for, rather than the exhaust of, emergent life. However, for this scenario to work requires the presence of sufficiently oxidizing electron acceptors.

Guided by results from geochemistry and extant biology (Mancinelli and McKay, 1988; Ducluzeau *et al.*, 2009; Ettwig *et al.*, 2010; Kampschreur *et al.*, 2011), we consider that nitric oxide, released on the partial reduction of the nitrogen oxyanions nitrate and nitrite in green rust (GR) (“fougerite” *sensu stricto*, Fe^II^_4_Fe^III^_2_(OH)_12_CO_3•_3H_2_O), is the most likely candidate for oxidizing methane in alkaline hydrothermal vent settings (Nitschke and Russell, 2013; Russell and Beckett, 2017). Again, in line with biology, we propose that methane was sequentially oxidized first to methanol and then to formaldehyde and maybe methylene. The view of a crucial role for nitrogen oxyanions and nitric oxide is given credence by estimations of NO pressure around a millibar at times in the Hadean atmosphere as in the work of Wong *et al.* (2017a)—enough to produce micromolar concentrations of nitrate and nitrite in the then ocean.

The fact that nitrate and nitrite can be reduced to NO with GR (Kampschreur *et al.*, 2011) provides the molecule that, in extant life, is known to serve as oxygen donor in the oxidative formation of methanol (Ettwig *et al.*, 2010). Counter-intuitively, the oxidation of the notoriously unreactive methane to methanol in biology requires a prior overreduction of the catalytic center binding both the methane and the oxygen donating molecule (O_2_ or NO). The redox potentials of the involved reactions are such that this prereduction can be performed either by electrons released from the subsequent oxidation steps (fed back as methanol is oxidized to formaldehyde and formaldehyde to methylene) or from hydrogen oxidation (Nitschke and Russell, 2013).

The reaction scheme both in biology and in our proposed scenario, therefore, is highly networked and contains numerous (autocatalytic) feedback loops (Fig. 1). We address the pertinence of this networking hereunder. The ultimately generated activated methyl is proposed to react with CO produced by the branch shown on the left-hand side of Figure 1. If, as we have proposed previously, during the emergence of life, this branch resembled that of extant organisms, then the electron donor to CO_2_ would be molecular hydrogen. However, the reduction of CO_2_ to CO by H_2_ is highly endergonic and involves the phenomenon of electron bifurcation in the respective organisms (Buckel and Thauer, 2013). Consequently, we have presumed that electron bifurcation may have played a corresponding role during life's emergence and that the two-electron redox metals molybdenum and tungsten might have been the primordial electron bifurcating agents (Nitschke and Russell, 2013). Recent results on the electrochemical properties of molybdopterin enzymes corroborate the possibility of such scenarios (Duval *et al.*, 2016).

**FIG. 2.**
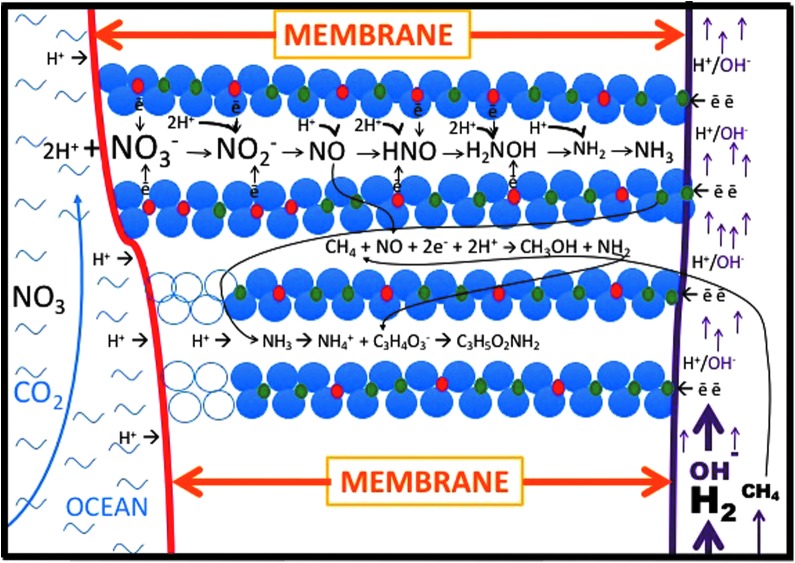
Model of GR as a ready-made difunctional enzyme precursor set in the inorganic membrane wherein it reduces nitrate to aminogen or ammonium between the “brucite” galleries (Trolard and Bourrié, 2012). At the same time, methane would be converted to a methyl group by NO (Kampschreur *et al.*, 2011)—a yet-to-be-tested hypothesis. Flores *et al.* (2016) showed that in these same circumstances, pyruvate can be aminated to alanine. The inorganic membrane or barrier, represented in this case by GR, separates the alkaline hydrothermal solution on the right, from ocean water on the left. Green rust was precipitated from the vast amounts of metastable iron precursors in the early oceans on meeting alkaline solutions such as those issuing from the vent (Arrhenius, 2003; Mielke *et al.*, 2010; Tosca *et al.*, 2016; Halevy *et al.*, 2017).

However, that carbon monoxide can also disproportionate from formate—known to be present in the alkaline vent fluids—under the slightly acidic conditions met with on the ocean side of the mound, adds a further (alternative or concomitant) source of CO to the system (Lang *et al.*, 2010). Formate-derived CO possibly provides the kick-starting for the two-pronged reaction scheme inherent in the Wood–Ljungdahl reactions common to methanotrophy, methanogenesis, and acetogenesis. The prize would be the provision of the ammonium ion for amination reactions within the reactor pile and associated chimneys through the concomitant reduction of the nitrate and/or nitrite (Fig. 2) (cf. Mancinelli and McKay, 1988; Haroon *et al.*, 2013; Arshad *et al.*, 2015).

## 3. The Pertinent Disequilibria (Free Energies) and Their Conversion

Baross and Hoffman (1985) foresaw that “a multiplicity of physical and chemical gradients” was to be expected “as a direct result of interactions between extensive hydrothermal activity in the Earth's crust and the overlying oceanic and atmospheric environments.” However, these gradients, especially pH and redox, would have been much more pronounced at putative submarine alkaline springs (Russell and Hall, 1997). There are two autotrophic free energy conversion mechanisms recognized as fundamental to driving life's endergonic operations, the proton motive force (pmf)/chemiosmosis to generate free energy for the cell (Mitchell, 1961, 1976; Boyer, 1975; Harold, 2001), and redox bifurcation whereby two electrons of the same energies may be released to single electron acceptors—one to a high potential acceptor and the other, “hot” electron, to reduce an otherwise well-defended low-potential acceptor (Baum *et al.*, 1967; Wikström and Berden, 1972; Mitchell, 1975, 1976; Kovacs, 1989; Iwata *et al.*, 1999; Thauer *et al.*, 2008; Nitschke and Russell, 2009, 2011, 2013; Kaster *et al.*, 2011; Chowdhury *et al.*, 2016; Peters *et al.*, 2016).

In the AVT, the emergence of the metabolic system was relieved of the necessity to pump protons out of the first compartments or protocells to generate the pmf, because a pre-existing steep proton gradient would have been imposed in the barriers from the acidulous Hadean ocean across to the alkaline interiors. Such a gradient is assumed, following the works of Baltscheffsky (1971), Baltscheffsky and Persson (2014), and Baltscheffsky *et al.* (1999), to have driven a pyrophosphatase, a primary ion pump with structure recently revealed by Kellosalo *et al.* (2012) and Tsai *et al.* (2014). We have appealed to GR as the flexible mineral precursor PPase to produce “the energy currency” of the cell (Lane, 2010; Branscomb and Russell, 2013; Russell *et al.*, 2013, fig. 5). Could the same mineral have played some part in the putative DMAP by oxidizing the methane to a methyl group in these hydrothermal conditions?

For the GR mineral, “fougerite,” to engineer the exergonic oxidation of methane to methanol—a kinetically highly challenging reaction given the stability of the methane molecule—requires an oxygen activated by adjacent ferrous irons within the double layers. Activated nitric oxide, formed as an intermediate in the reduction of nitrate or nitrite to ammonium within the galleries of GR, is the likely candidate (Hansen *et al.*, 2001; Ducluzeau *et al.*, 2009; Kampschreur *et al.*, 2011; Wu *et al.*, 2015; Wong *et al.*, 2017a). Further indications that GR could act as a potential mediator of such a reaction are (1) because of its structural similarity to the metal cofactor of soluble methane monooxygenase and (2) for its variable valence (Nitschke and Russell, 2013; Nitschke *et al.*, 2013; Banerjee *et al.*, 2015). Moreover, the two oxygen atoms forming the diamond center are both derived from O_2_ (Banerjee *et al.*, 2015). However, as nitric oxide is isoelectric with dioxygen and, moreover, is slightly asymmetric, and has similar interaction properties as O_2_ (Pilet *et al.*, 2004), it is potentially as strong a candidate as oxygen to activate the methane bound to the metal site for oxidation.

**FIG. 3.**
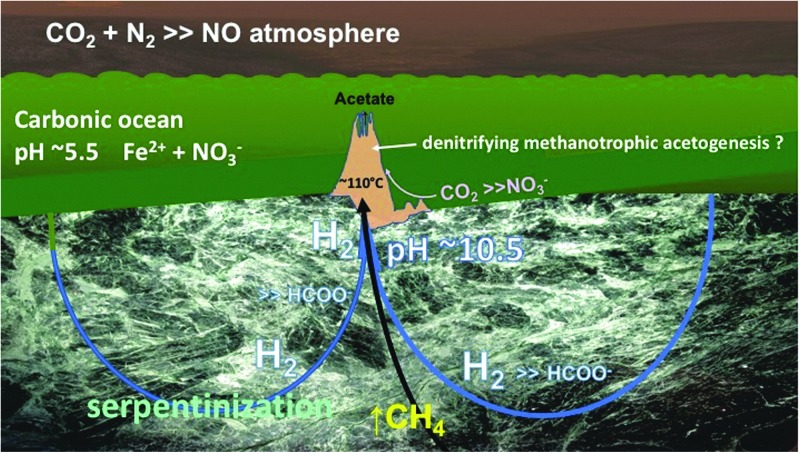
Schematic representation of alkaline hydrothermal vent model for the early emergence of life on Earth through a putative denitrifying methanotrophic acetogenic pathway fed from H_2_ generated through serpentinization while methane is leached from ambient reduced carbon molecules residing in the crust (Russell and Hall, 1997; Proskurowski *et al.*, 2008; Nitschke and Russell, 2013).

Although GR itself has never been shown to oxidize methane to methanol, a bent mono(μ-oxo)di-nickel anchored within a synthetic zeolite does promote such an oxidation at 150°C (Shan *et al.*, 2014; and see Starokon, *et al.*, 2013). Clearly, a demonstration of methane oxidation by nitric oxide intercalated within GR or other iron-rich double layer hydroxide (DLH) in hydrothermal conditions must be demonstrated for the denitrifying methanotrophic acetogenesis hypothesis to survive (Figs. 2 and 3).

As mentioned, furnishing CO from CO_2_ in the left-hand branch of Figure 1 is highly endergonic and thus requires redox bifurcation to occur. For this to happen, a molybdenum atom (or atoms) would then need to be hosted in the DLH (Nitschke and Russell, 2009, 2013). We have speculated that a molybdenum sulfide complex [FeO(OH)(MoS_4_)_2_]^3−^ (Helz *et al.*, 2014), sequestered in the interlayers of GR or other DLH, would be secured through hydrogen bonding, much as an H-bond network surrounding the pyranopterins in the molybdenum site modulates two-electron redox properties in arsenite oxidase (Itaya *et al.*, 1987; Russell *et al.*, 2014; Duval *et al.*, 2016).

## 4. Astrobiological Implications

That methane concentrations at alkaline vents and volcanic eruptions are so low on Earth is because of the surprisingly high oxidation state of Earth's mantle throughout geological history; this is surprising because the planetesimals and meteorites from which it accumulated are generally buffered below iron-wustite and contain reduced organic compounds (Shock, 1992; McSween and Huss, 2010; Dale *et al.*, 2012; Mousis *et al.*, 2015). Wood *et al.* (2006) explained this relatively high state by recognizing the tendency of ferrous iron (mainly in olivine) to disproportionate in the presence of perovskite which is stable at depths below the 660-km discontinuity, that is, at 24 GPa and 1900 K (McCammon, 1997; Wood, 2000; Chudinovskikh and Boehler, 2001; Frost *et al.*, 2004). The mantles of smaller bodies would be at much lower pressure and, therefore, more reduced (Wadhwa, 2008; Dale *et al.*, 2012; Gaillard *et al.*, 2015). The resultant low-oxygen fugacities would explain the preponderance of methane on some of the moons of Saturn (Waite *et al.*, 2009; Bouquet *et al.*, 2015; Glein *et al.*, 2015; Dorofeeva, 2016), possibly Europa (Goodman *et al.*, 2004; Hand *et al.*, 2007; Zolotov and Kargel, 2009) and, perhaps, even early Mars (Wadhwa, 2001; Hirschmann and Withers, 2008; McSween *et al.*, 2009; Blamey *et al.*, 2015; Edwards and Ehlmann, 2015; Hu *et al.*, 2015).

As with the early Earth, the icy moons would, while producing copious reductants, soon face an oxidant crisis. Denitrifying methanotrophic acetogenesis might well be the emergent metabolism on such worlds, with nitrate/nitrite produced in the ice shell through the oxidation of ammonia with hydrogen peroxide entrained from the surface as electron acceptors (Loeffler and Hudson, 2015; Russell *et al.*, 2017).

## 5. Discussion and Issues Raised

Our main aim of this work is to reconsider our previous expectations of a relationship between the geochemistry of moderate temperature serpentinization with that of early biochemical pathways, namely, was biochemical methanogenesis derived from a rapid and facile geochemical reduction of CO_2_ to CH_4_, that is, from abiotic methanogenesis (Martin and Russell, 2007)? It was reasoned that such a reaction had to be rapid enough compared with the product dissipation rate to maintain a self-organizing protometabolic pathway and support continued growth. In other words, it would need to happen within minutes to hours if it were to be the harbinger of enzyme-catalyzed reactions that are generally measured in milli- to microseconds or less (Garrett and Grisham, 2012, page 17).

It follows that the results reported from various laboratories and field sites, which we accept, militate against the idea that biochemical methanogenesis emerged partly through a *quickening* of geochemical reactions. Moreover, the thermodynamic calculations of Shibuya *et al.* (2016) estimate the delivery of formaldehyde and acetate from hydrothermal systems generated by serpentinization to be 9.7 × 10^−27^ and 8.6 × 10^−24^ molal, respectively. Formaldehyde, which anyway can be difficult to analyze and is a significant contaminant, has not been registered in serpentinization experiments (Borowska and Mauzerall, 1991; Barro *et al.*, 2009). And Lang *et al.* (2010), analyzing acetate at Lost City, found concentrations between 1 and 35 μmol/kg for which they tentatively assumed a microbial derivation.

Another point at issue is whether hydrogen was the main fuel as we and others have claimed, or whether hydrothermal formate fulfilled that role as suggested by Windman *et al.* (2007). As mentioned, formate will disproportionate to CO and water on being driven through the inorganic hydrothermal mound toward the acidulous ocean. CO is then available to react with the methyl group produced through the supposed oxidation of methane as already alluded. An alternative, or early derivation, of this tributary to the DMAP would be a reverse of the formate hydrogenlyase (FHL) reaction (Andrews, 1997), whereby the proton gradient (acidulous ocean juxtaposed to the alkaline, hydrogen-bearing interior) would drive the generation of formate from CO_2_ with the two electrons supplied from H_2_ or HCOO^−^ through a molybdenum-dosed nickel-rich mackinawite (FeNiS_2_)_n_ or greigite ([FeNi]_3_S_4_)_n_ nanocluster that acts as a hydrogen store and protohydrogenase (Cao *et al.*, 2009; Nitschke and Russell, 2009, 2013; Bassegoda *et al.*, 2014; Yamaguchi *et al.*, 2014; Wang *et al.*, 2015; White *et al.*, 2015; Wilkin and Beak, 2017). Pinske and Sargent (2016) demonstrated unambiguously that FHL enzyme is bidirectional, so giving some strength to this hypothesis (and see Roger *et al.*, 2017).

A theoretical assessment of whether molybdenum-bearing GR can act as the engine to drive the DMAP would require *ab initio* molecular dynamic simulations to indicate how redox changes in GR involving electron and proton transport compare with methane monooxygenase. From our ideas about the quickening of abiotic serpentinization reductions, it seems imaginable that such a rapid and facile reduction of CO_2_ to HCOO^−^ could be taken over by emerging life as one step in carbon fixation. Nevertheless, these results also threaten the facile view that hydrogen was the very first fuel for life, that is, the electron donor in a redox chain that can then drive other chemistry (Windman *et al.*, 2007). Contrarily, it is extremely unlikely that CO_2_ was hydrogenated directly to methane. ^12^CH_4_ is presumably distilled, or cracked, from organic molecules previously lodged in crystalline and sulfidic source rocks (Strauss, 1989). So, although it had been commonly considered that archaeal methanogens—exploiting or even discovering the reductive acetyl-coA pathway—occupied the very lowest branches of the evolutionary tree, recent experiments show that this idea can no longer be entertained (cf., Koonin and Martin, 2005; Russell *et al.*, 2005; Martin and Russell, 2007; Wolfe, 2014; Shock and Boyd, 2015).

At lower redox and pH, there is a transition from HCOO^−^ to CO (Keene, 1993). Huber and Wächtershäuser (1997) showed how, given a methane thiol, acetate can be generated by the comparable Monsanto reaction (Crabtree, 1977). Huber and Wächtershäuser (1997) called upon a mechanism proposed in an article by Heinen and Lauwers (1996) to supply the methyl group, supposedly generated through the reduction of CO_2_ in water in the presence of H_2_S and FeS at low pH. However, the yields are ∼0.1% or less of the available H_2_S (as HS^−^), which itself is only one millimole (and see Schoonen *et al.*, 1999 for a critique). So even supposing all the hydrogen sulfide is converted to methyl sulfide, this would provide <1 nmol/L—hardly enough to satisfy the ∼50 mmol/L of formate to produce activated acetate.

The only way for some form of the acetyl-coA pathway to survive as the first carbon fixation hypothesis is for the methyl group to be provided from a different origin. Alert to this dilemma, it has been speculated that the necessary methyl group was provided by the oxidation of the juvenile methane by Fe^3+^-rich GR at the alkaline hydrothermal mound (Nitschke and Russell, 2013; Russell *et al.*, 2014; cf., Scheller *et al.*, 2016). This explanation is amenable to experimental falsification (cf., Diaz-Campos *et al.*, 2009). Wu *et al.* (2013) demonstrated the direct synthesis of acetate from CO_2_ and methane on a zinc-modified aluminosilicate zeolite, and the idea gleans some further support in that methane can be oxidized to methanol at 160°C on a synthetic iron-bearing zeolite previously calcined in the presence of nitrous oxide at 200–250°C (Starokon *et al.*, 2013).

As we have seen, the initially assumed hydrogenation of CO_2_ to methane by H_2_, proposed to then segue into methanogenic metabolism, probably does not occur at significant velocities and yields. Moreover, it is also comparatively poorly suited for rationalizing life's emergence in the framework of the laws of the physical world. As pointed out half a century ago (Schrödinger, 1944), the second law of thermodynamics imposes the requirement that life, and *a fortiori* its emergence, is possible only as a subset of a larger system featuring strong thermodynamic disequilibria. The formidable entropy decrease inherent in living things can thus be fueled by low-entropy sources from their environment. The entropy of the entire system will then increase despite a local decrease in the living subset.

Although the second law (Boltzmann's entropy law) specifies under which conditions life is, or is not, possible, it does not provide a mechanism to rationalize its emergence. However, recent developments achieved, and the insights gained, within the field of far-from-equilibrium thermodynamics do indicate the kind of mechanism required to bring about the onset of life (Branscomb and Russell, 2013; Branscomb *et al*., 2017). Systems far from thermodynamic equilibrium have indeed been shown to frequently generate substantially ordered and self-organizing states as they relax toward equilibrium, that is, while dissipating free energy (Prigogine and Nicolis, 1967, 1989).

The fields of rheology, atmospheric sciences, or networked chemical reactions are abound with examples of such “dissipative structures.” These feature many of the defining properties of living systems, and the emergence of a dissipative structure, “life,” under appropriate conditions of high disequilibria appears as a quasi-necessity in the theoretical framework of far-from-equilibrium thermodynamics. In addition to strong free energy gradients characterizing the system, far-from-equilibrium thermodynamics has worked out further important parameters that favor the emergence of dissipative structures: (a) nonlinear equations of motion in reaction phase space and (b) reaction feedback loops (note that mathematically (a) and (b) are often intimately correlated with the high ΔG criterion).

How do the two alternative hypothetical founding metabolic reactions in which methane appears either as an exhaust (methanogenesis) or as a fuel (methanotrophy) fare against the test of the already listed criteria? The reaction hydrogenating CO_2_ to methane certainly features a substantial negative ΔG under most conditions (*i*.*e*., concentrations of reactants and pH values) likely encountered in alkaline hydrothermal vents of the Hadean (Amend *et al.*, 2013). However, this is the total ΔG of the bulk solution, and its high value stems from the significant concentrations of reactants in the reaction mixture.

The individual redox reaction (4H_2_ + CO_2_ ↔ CH_4_ + 2H_2_O), by contrast, is, under standard conditions, among the least exergonic (ΔE_m_ of about 150 mV, N.B.: ΔG^o^–nFΔE_m_) free energy converting reactions exploited by life (Schoepp-Cothenet *et al.*, 2013). That this reaction actually is close to equilibrium is nicely illustrated by the fact that the sequence of redox transitions operating in methanogens in the presence of H_2_ and CO_2_, but at low concentrations of CH_4_, is readily reversed in methanotrophic Archaea when CH_4_ concentrations become significant (Haroon *et al.*, 2013).

More importantly, both the biological and the posited abiotic reduction of CO_2_ to methane (Martin and Russell, 2007) proceed through sequential and mutually independent electron donations, in the latter case to, for example, the reduced carbonaceous chondrites (the C1-stony meteorites). From the point of view of far-from-equilibrium thermodynamics, the absence of feedback loops necessary for inducing autocatalysis and hence self-amplification during the approach to equilibrium of a system that was not far from equilibrium to begin with, *a priori*, does not look favorable for generating a dissipative structure.

The proposed natural pH of alkaline hydrothermal vents (Russell *et al.*, 1989) as well as the recently discovered (Thauer *et al.*, 2008; Chowdhury *et al.*, 2016) flavin-based electron bifurcation reaction, however, is quintessential to entropy-decreasing engines (Cottrell, 1979; Branscomb and Russell, 2013) and, therefore, adds some of the ingredients required for the emergence of self-organizing systems to the reaction scheme advocated by Lane and Martin (2012 and see *e.g.,* Weiss *et al.*, 2016).

Methane-oxidizing reactions, such as, for example, that which is pulled by nitrogen oxides and oxyanions, yield a completely different picture (Fig. 1). The drop in free energy pertaining to their individual redox reactions (ΔE_m_) exceeds 1 V (Schoepp-Cothenet *et al.*, 2013). Furthermore, they are replete with feedback loops, both at the level of substrates (the nitrogen compounds) and of reducing equivalents (Fig. 1). Since subsequent one-electron oxidation states of methane feature increasingly reducing electrochemical potentials, these can feed back into the initial reactions of methane activation and of the reduction of nitrogen oxyanions. Obviously, the already mentioned entropy-lowering engines employing the pH gradient and of electron bifurcation are expected to also play crucial roles in the scenario of denitrifying methanotrophy (Nitschke and Russell, 2013).

From the already described observations, we conclude that a reaction producing methane as an exhaust does not only appear geochemically compromised but also comparatively poorly suited (although not excluded) for kick-starting life's emergence in the form of a dissipative structure. By contrast, the oxidation of the “fuel” methane by high potential electron acceptors, such as, for example, nitrogen oxides and oxyanions, fulfills several of the criteria laid out by far-from-equilibrium thermodynamics for the emergence of self-organizing systems. A major task for future research into the details of a putative emergence of life driven by denitrifying methanotrophic acetogenesis will be to work out the behavior of reactants and products under different ranges of initial conditions.

## 6. Résumé and Conclusions

As CO_2_ may be rapidly hydrogenated to formate, it seems plausible that this reduction was taken over by emerging life as one step in carbon fixation (Seewald *et al.*, 2006; Lang *et al.*, 2010, 2012; Nitschke and Russell, 2013). However, a rapid and total hydrogenation of CO_2_ to methane is slow and does not produce the necessary metastable intermediates (McCollom and Seewald, 2003). Most of the CH_4_ at Lost City and other alkaline springs is presumably distilled, or cracked, from organic molecules previously lodged in crystalline and sulfidic source rocks (Strauss, 1989; Proskurowski *et al.*, 2008). This finding is a stumbling block to the acetyl-coA pathway being the first metabolic pathway as negotiated by the methanogens. We, and others, had hitherto considered these archaea to occupy the lowest branches of the evolutionary tree (Koonin and Martin, 2005; Russell *et al.*, 2005; Martin and Russell, 2007; Wolfe, 2014; Shock and Boyd, 2015).

So the serpentinization reaction appears limited to the direct delivery of the fuels H_2_ and, perhaps, HCOO^−^/CO—reduced from CO_2_—to emergent life, whereas the provision of CH_4_ is through the processing of pre-existing carbonaceous components or by entrainment of the volatile residing, or produced in, the oceanic lithosphere (Watanabe *et al.*, 1983; Pineau and Mathez, 1990; Seewald *et al.*, 2006; Proskurowski *et al.*, 2008; Lazar *et al.*, 2012, 2015; Paukert *et al.*, 2012; McCollom and Seewald, 2013; Lollar *et al.*, 2014; Suda *et al.*, 2014; McDermott *et al.*, 2015; Seyfried *et al.*, 2015; McCollom and Donaldson, 2016).

In detail, we conclude from our critical review:

1. The serpentinization reaction—sometimes alluded to as the “sister” of early metabolism—does not reduce carbon dioxide to methane, nor to methane thiol, at least not rapidly enough for it to be thought of as a precursor to a metabolic reaction (Fig. 3) (Reeves *et al.*, 2014; McCollom and Donaldson, 2016). As we have tried to convey in this article, serpentinization likely was the “mother engine” of life—not its sister (Russell *et al.*, 2013)! Although the serpentinization reaction reduces some of the interacting circulating aqueous fluid to hydrogen, perhaps over a period of hundreds to thousands of years, that reduction too is neither facile nor rapid (McCollom and Donaldson, 2016).

These experimental findings contradict expectations that biochemistry first emerged as parallels to serpentinization reactions, that is, directly from geochemistry (cf., Russell and Hall., 1997; Martin and Russell, 2007; Nitschke and Russell, 2009). Lang *et al.* (2010) also cast very considerable doubt on the view that acetate may have been an immediate product of serpentinization. These authors found acetate at Lost City to vary nonsystematically with hydrothermal-to-ocean fluid chemistry from 1 to 35 μmol/kg for which they tentatively assumed a microbial derivation. We note also that the calculations of Shibuya *et al.* (2016) put acetate in Hadean hydrothermal springs at ∼10^−23^ molal.

In contrast, reduction of CO_2_ to formate *is* rapid during serpentinization (timescale of minutes or tens of minutes according to White (2013) and Herschy *et al.* (2014)). Also, Lang *et al.* (2010) recorded ∼150 μmol/kg of formate in the end-member hydrothermal fluids issuing from Lost City (and see McCollom and Seewald, 2001, 2003; McDermott *et al.*, 2015). The serpentinization reaction thus appears limited to the direct delivery of HCOO^−^ (reduced from CO_2_) to emergent life. These results force a rethink of the idea that the classic acetyl coenzyme pathway was the harbinger of life (Russell and Hall, 1997; Russell and Martin, 2004). In this regard, we note that Windman *et al.* (2007) suggested formate as an alternative source of fuel *and* reduced carbon, in place of H_2_ and CO_2_.

2. In our view, a modified (oxidative) version of the acetyl-coA pathway survives as the first pathway to life, although requiring nitrate and/or nitrite in early oceans as an electron acceptor to oxidize iron in GR as well as hydrothermal methane (Fig. 1). This hypothesized earliest metabolism has been termed “denitrifying methanotrophic acetogenesis” (Nitschke and Russell, 2013; Stern *et al.*, 2015; Wong *et al.*, 2017a). Although there has been some tenuous experimental support for this hypothesis (Russell *et al.* 2014 and references therein), the conclusion is again forced that an inorganic membrane is required to provide the vectorial redox and pH gradients (chemiosmotic disequilibrium) to drive life's emergence (Russell *et al.*, 1989, 1994) in line with the works of Herschy *et al.* (2014) and Sojo *et al.* (2015) (and see Batista and Steinbock, 2015 for future experimental approaches).

In particular, Herschy *et al.* (2014) demonstrated that a proton gradient acting across thin nickel-doped mackinawite ([Fe>>Ni]S) membranes separating hot (70°C) alkaline (pH 11) solution from an ancient ocean simulant ∼pH 5 and ∼20°C appears also to drive the reduction of CO_2_ to formate (∼50 μmol/L), although not to acetate. We conclude that dispositive experimentation must demonstrate that nitric oxide, produced from nitrite/nitrate and activated in the interlayers of an Fe^2+^-rich GR, is called for as oxidant before the DMAP hypothesis could be acceptable (McGlynn *et al.*, 2009; Nitschke and Russell, 2013). To carry out such tests, we envision juxtaposing a cool carbonate/nitrate/nitrite-bearing early acidulous ocean simulant, across the precipitate membrane, to an alkaline solution of hydrogen and methane in a high-pressure hydrothermal reaction chamber.

3. Our general conclusion is that what drove life's emergence was not merely speeding up of chemistry or geochemistry (toward biochemistry) as sometimes assumed. Key to the emergence and the maintenance of all life are specific enzymes, many of which are effectively disequilibria-converting engines (Branscomb and Russell, 2013; Branscomb *et al.*, 2017). These are turnstile-like engines, often housed in membranes that, for example, couple strong redox and pH gradients to drive endergonic reactions (Branscomb and Russell, 2013; Branscomb *et al.*, 2017). Reaction–diffusion systems are another mechanism for driving disequilibria conversions at the submarine alkaline vent (Epstein and Xu, 2016).

Comprising portions of the membrane and the exhalative pile, GR offers a chemically active, and confining, matrix that is not that different from the construct of a cell in which the several potential reactants could compete between diffusion distances and reaction rates, resulting in the self-organization of products as indicated in Figure 2 (Russell *et al.*, 2003; Russell and Beckett, 2017; and see Johnson *et al.*, 2015; Forticaux *et al.*, 2015; Johannessen *et al.*, 2016). A vital requirement for emergence-of-life research is to discover and demonstrate the power of minerals to act as such drivers and facilitators through flexuring, chemical waves, and changes of conformation, effected through redox, acid–base, and hydrolysis reactions (Arrhenius, 2003; Coveney *et al.*, 2012; Hoffmann, 2012; Russell *et al.*, 2013; Branscomb *et al.*, 2016; Epstein and Xu, 2016).

4. With the proviso that “(E)xothermic serpentinization of ocean crust is life's mother engine” (Russell *et al.*, 2013), we entertain the view that, far from being one of the first waste products of metabolism, abiotic methane was likely a fuel of methanotrophy (Evans *et al.*, 2015 and supplementary information). On reflection, it is certainly more logical to see methane as a reductant, one, along with hydrogen—for it to be partially oxidized—thereby providing another portion of the carbon to the first biotic organic molecules (Ducluzeau *et al.*, 2009, 2014; Nitschke and Russell, 2013).

5. An astrobiological implication: That the wet and rocky bodies in the solar system smaller than Earth probably have little or no bridgmanite (perovskite) and, therefore, have more reduced mantles may explain the preponderance of methane on some of the moons of Saturn (Wood *et al.*, 2006; Bouquet *et al.*, 2015; Dorofeeva, 2016; Girard *et al.*, 2016; Gu *et al.*, 2016), possibly Europa (Zolotov and Kargel, 2009) and even early Mars (Wadhwa, 2001; Edwards and Ehlmann, 2015; Hu *et al.*, 2015; Wong *et al.*, 2017b). The challenge then is to consider the availability of electron acceptors on these other worlds (*e.g.*, Nealson, 1997; Russell *et al.*, 2017).

## Abbreviations Used

AVT: alkaline vent theory
DLH: double layer hydroxide
DMAP: denitrifying methanotrophic acetogenic pathway
FHL: formate hydrogenlyase
GR: green rust
pmf: proton motive force

## References

[B1] Abrajano T.A., Sturchio N.C., Bohlke J.K., Lyon G.L., Poreda R.J., and Stevens C.M. (1988) Methane-hydrogen gas seeps, Zambales Ophiolite, Philippines: deep or shallow origin? Chem Geol 71:211–222

[B2] Amend J.P. and Shock E.L. (2001) Energetics of overall metabolic reactions of thermophilic and hyperthermophilic Archaea and Bacteria. FEMS Microbiol Rev 25:175–243 11250035 10.1111/j.1574-6976.2001.tb00576.x

[B3] Amend J.P., LaRowe D.E., McCollom T.M., and Shock E.L. (2013) The energetics of organic synthesis inside and outside the cell. Phil Trans R Soc B 368:20120255 23754809 10.1098/rstb.2012.0255 PMC3685458

[B4] Arrhenius G.O. (2003) Crystals and life. Helv Chim Acta 86:1569–1586

[B5] Arshad A., Speth D.R., de Graaf R.M., den Camp H.J.O., Jetten M.S., and Welte C.U. (2015) A metagenomics-based metabolic model of nitrate-dependent anaerobic oxidation of methane by *Methanoperedens*-like Archaea. Front Microbiol 6:1423 26733968 10.3389/fmicb.2015.01423 PMC4683180

[B6] Baltscheffsky H. (1971) Inorganic pyrophosphate and the origin and evolution of biological energy transformation (biological energy transformation origin and evolution, dis-cussing inorganic pyrophosphates precursor to adenosine phosphates as energy carriers). In Chemical Evolution and the Origin of Life, edited by Buvet R. and Ponnamperuma C., North-Holland Pub. Cy., Amsterdam, pp 466–474

[B7] Baltscheffsky H. and Persson B. (2014) On an early gene for membrane-integral inorganic pyrophosphatase in the genome of an apparently pre-LUCA extremophile, the archaeon *Candidatus Korarchaeum cryptofilum*. J Mol Evol 78:140–147 24477828 10.1007/s00239-014-9610-7

[B8] Baltscheffsky M., Schultz A., and Baltscheffsky H. (1999) H^+^-PPases: a tightly membrane-bound family. FEBS Lett 457:527–533 10523139 10.1016/s0014-5793(99)90617-8

[B9] Banerjee R., Proshlyakov Y., Lipscomb J.D., and Proshlyakov D.A. (2015) Structure of the key species in the enzymatic oxidation of methane to methanol. Nature 518:431–434 25607364 10.1038/nature14160 PMC4429310

[B10] Baross J.A. and Hoffman S.E. (1985) Submarine hydrothermal vents and associated gradient environments as sites for the origin and evolution of life. Orig Life Evol Biosph 15:327–345

[B11] Barro R., Regueiro J., Llompart M., and Garcia-Jares C. (2009) Analysis of industrial contaminants in indoor air: Part 1. Volatile organic compounds, carbonyl compounds, polycyclic aromatic hydrocarbons and polychlorinated biphenyls. J Chromat A 1216:540–566 10.1016/j.chroma.2008.10.117 19019381

[B12] Bassegoda A., Madden C., Wakerley D.W., Reisner E., and Hirst J. (2014) Reversible interconversion of CO_2_ and formate by a molybdenum-containing formate dehydrogenase. J Am Chem Soc 136:15473–15476 25325406 10.1021/ja508647u

[B13] Batista B.C. and Steinbock O. (2015) Growing inorganic membranes in microfluidic devices: chemical gardens reduced to linear walls. J Phys Chem C 119:27045–27052

[B14] Baum H., Rieske J.S., Silman H.I., and Lipton S.H. (1967) On the mechanism of electron transfer in complex III of the electron transfer chain. Proc Natl Acad Sci USA 57:798–805 16591533 10.1073/pnas.57.3.798 PMC335578

[B15] Blamey N.J., Parnell J., McMahon S., Mark D.F., Tomkinson T., Lee M., Shivak J., Izawa M.R.M., Banerjee N.R., and Flemming R.L. (2015) Evidence for methane in Martian meteorites. Nat Commun 6:7399, doi:10.1038/ncomms8399 26079798 PMC4521231

[B16] Borowska Z. and Mauzerall D. (1991) Retraction. “Photoreduction of carbon dioxide by aqueous ferrous ion: an alternative to the strongly reducing atmosphere for the chemical origin of life.” Proc Natl Acad Sci USA 88:4564 16593977 10.1073/pnas.85.18.6577 PMC282019

[B17] Bouquet A., Mousis O., Waite J.H., and Picaud S. (2015) Possible evidence for a methane source in Enceladus' ocean. Geophys Res Lett 42:1334–1339

[B18] Boyer P.D. (1975) A model for conformational coupling of membrane potential and proton translocation to ATP synthesis and to active transport. FEBS Lett 58:1–6 1225567 10.1016/0014-5793(75)80212-2

[B19] Branscomb E. and Russell M.J. (2013) Turnstiles and bifurcators: the disequilibrium converting engines that put metabolism on the road. Biochim Biophys Acta 1827:62–78 23063910 10.1016/j.bbabio.2012.10.003

[B20] Branscomb E., Biancalani T., Goldenfeld N., and Russell M.J. (2017) Escapement mechanisms and the conversion of disequilibria: the engines of creation. Phys Rep 677:1–60

[B21] Cao F., Hu W., Zhou L., Shi W., Song S., Lei Y., Wang S., and Zhang H. (2009) 3D Fe_3_S_4_ flower-like microspheres: high-yield synthesis via a biomolecule-assisted solution approach, their electrical, magnetic and electrochemical hydrogen storage properties. Dalton Trans 9246–9252 20449202 10.1039/b912569h

[B22] Chistoserdova L., Kalyuzhnaya M.G., and Lidstrom M.E. (2009) The expanding world of methylotrophic metabolism. Ann Rev Microbiol 63:477–499 19514844 10.1146/annurev.micro.091208.073600 PMC2827926

[B23] Chowdhury N.P., Klomann K., Seubert A., and Buckel W. (2016) Reduction of flavodoxin by electron bifurcation and sodium ion-dependent reoxidation by NAD^+^ catalyzed by ferredoxin-NAD^+^ reductase (Rnf). J Biol Chem 291:11993–12002 27048649 10.1074/jbc.M116.726299 PMC4933252

[B24] Chudinovskikh L. and Boehler R. (2001) High-pressure polymorphs of olivine and the 660-km seismic discontinuity. Nature 411:574–577 11385569 10.1038/35079060

[B25] Ciccarelli F.D., Doerks T., Von Mering C., Creevey C.J., Snel B., and Bork P. (2006) Toward automatic reconstruction of a highly resolved tree of life. Science 311:1283–1287 16513982 10.1126/science.1123061

[B26] Corliss J.B., Baross J.A., and Hoffman S.E. (1981) An hypothesis concerning the relationships between submarine hot springs and the origin of life on earth. Oceanologica Acta 4 (suppl): 59–69

[B27] Cottrell A. (1979) The natural philosophy of engines. Contemp Phys 20:1–10

[B28] Coveney P.V., Swadling J.B., Wattis J.A., and Greenwell H.C. (2012) Theory, modelling and simulation in origins of life studies. Chem Soc Rev 41:5430–5446 22677708 10.1039/c2cs35018a

[B29] Coveney R.M., Geobel E.D., Zeller E.J., Dreschhoff G.A.M., and Angino E.E. (1987) Serpentinization and the origin of hydrogen gas in Kansas. Bull Am Assoc Petrol Geol 71:39–48

[B30] Crabtree R.H. (1997) Where smokers rule. Science 276:222 9132945 10.1126/science.276.5310.222

[B31] Dale C.W., Burton K.W., Greenwood R.C., Gannoun A., Wade J., Wood B.J., and Pearson D.G. (2012) Late accretion on the earliest planetesimals revealed by the highly siderophile elements. Science 336:72–75 22491852 10.1126/science.1214967

[B32] Darwin F.E. (1888) The Life and Letters of Charles Darwin. John Murray, London

[B33] Degens E.T. (1979) Primordial synthesis of organic matter. In The Global Carbon Cycle. edited by Bolin B., Degens E.T., Kempe S., and Ketner P., John Wiley, New York, pp 57–77

[B34] Diaz-Campos M., Akkutlu I.Y., and Sigal R.F. (2009) A molecular dynamics study on natural gas solubility enhancement in water confined to small pores. Soc Pet Eng 124491:1–10

[B35] Dorofeeva V.A. (2016). Genesis of volatile components at Saturn's regular satellites. Origin of Titan's atmosphere. Geochem Int 54:7–26

[B36] Ducluzeau A.L., Schoepp-Cothenet B., Baymann F., Russell M.J., and Nitschke W. (2014) Free energy conversion in the LUCA: quo vadis? Biochim Biophys Acta 1837:982–988 24361840 10.1016/j.bbabio.2013.12.005

[B37] Ducluzeau A.-L., van Lis R., Duval S., Schoepp-Cothenet B., Russell M.J., and Nitschke W. (2009) Was nitric oxide the first strongly oxidizing terminal electron sink? Trends Biochem Sci 34:9–15 19008107 10.1016/j.tibs.2008.10.005

[B38] Duval S., Santini J.M., Lemaire D., Chaspoul F., Russell M.J., Grimaldi S., Nitschke W., and Schoepp-Cothenet B. (2016) The H-bond network surrounding the pyranopterins modulates redox cooperativity in the molybdenum-bisPGD cofactor in arsenite oxidase. Biochim Biophys Acta 1857:1353–1362 27207587 10.1016/j.bbabio.2016.05.003

[B39] Edwards C.S., and Ehlmann B.L. (2015) Carbon sequestration on Mars. Geology 43:863–866

[B40] Epstein I.R., and Xu B. (2016) Reaction-diffusion processes at the nano-and microscales. Nat Nanotech 11:312–319 10.1038/nnano.2016.41 27045215

[B41] Etiope G., Ehlmann B.L., and Schoell M. (2012) Low temperature production and exhalation of methanefrom serpentinized rocks on Earth: a potential analog for methane production on Mars. Icarus 224, doi:10.1016/j.icarus.2012.05.009

[B42] Ettwig K.F., Butler M.K., Le Paslier D., Pelletier E., Mangenot S., Kuypers M.M.M., Schreiber F., Dutilh B.E., Zedelius J., de Beer D., Gloerich J., Wessels H.J.C.T. van Alen T., Luesken F., Wu M.L., van de Pas-Schoonen K.T., Op den Camp H.J.M., Janssen-Megens E.M., Francoijs K.-J., Stunnenberg H., Weissenbach J., Jetten M.S.M., and Strous M. (2010) Nitrite-driven anaerobic methane oxidation by oxygenic bacteria. Nature 464:543–548 20336137 10.1038/nature08883

[B43] Evans P.N., Parks D.H., Chadwick G.L., Robbins S.J., Orphan V.J., Golding S.D., and Tyson G.W. (2015) Methane metabolism in the archaeal phylum Bathyarchaeota revealed by genome-centric metagenomics. Science 350:434–438 26494757 10.1126/science.aac7745

[B44] Flores E., Barge L., VanderVelde D., Kallas K., Baum M.M., Russell M.J., and Kanik I. (2016) Amino acid synthesis in seafloor environments on icy worlds. Am Astron Soc DPS Meeting #48, id.#323.02

[B45] Forticaux A., Dang L., Liang H., and Jin S. (2015) Controlled synthesis of layered double hydroxide nanoplates driven by screw dislocations. Nano Lett 15:3403–3409 25870920 10.1021/acs.nanolett.5b00758

[B46] Frost D.J., Liebske C., Langenhorst F., McCammon C.A., Trønnes R.G., and Rubie D.C. (2004) Experimental evidence for the existence of iron-rich metal in the Earth's lower mantle. Nature 428:409–412 15042086 10.1038/nature02413

[B47] Gaillard F., Scaillet B., Pichavant M., and Iacono-Marziano G. (2015) The redox geodynamics linking basalts and their mantle sources through space and time. Chem Geol 418:217–233

[B48] Garrett R. and Grisham C. (2012) Biochemistry. Nelson Education, Toronto

[B49] Génin J.-M.R., Guérin O., Herbillon A.J., Kuzman E., Mills S.J., Morin G., Ona-Nguema G., Ruby C. and Upadhyay C. (2012) Redox topotactic reactions in Fe^II-III^ (oxy)hydroxycarbonate new minerals related to fougerite in gleysols; “trébeurdenite” and “mössbauerite”. In ICAME 2011. Springer, Netherlands, pp 71–81

[B50] Girard J., Amulele G., Farla R., Mohiuddin A., and Karato S.I. (2015) Shear deformation of bridgmanite and magnesiowüstite aggregates at lower mantle conditions. Science 351:144–147 26721681 10.1126/science.aad3113

[B51] Glein C.R. (2015) Noble gases, nitrogen, and methane from the deep interior to the atmosphere of Titan. Icarus 250:570–586

[B52] Goldschmidt V.M. (1952) Geochemical aspects of the origin of complex organic molecules on Earth, as precursors to organic life. New Biol 12:97–105

[B53] Goodman J.C., Collins G.C., Marshall J., and Pierrehumbert R.T. (2004) Hydrothermal plume dynamics on Europa: implications for chaos formation. J Geophys Res Planets 109: E03008, doi: 10.1029/2003JE002073

[B54] Greenberger R.N., Mustard J.F., Cloutis E.A., Pratt L.M., Sauer P.E., Mann P., Turner K., Dyar M.D., and Bish D.L. (2015) Serpentinization, iron oxidation, and aqueous conditions in an ophiolite: implications for hydrogen production and habitability on Mars. Earth Planet Sci Lett 416:21–34

[B55] Gu T., Li M., McCammon C., and Lee K.K. (2016) Redox-induced lower mantle density contrast and effect on mantle structure and primitive oxygen. Nat Geosci 9:723–727

[B56] Haeckel E. (1892) The History of Creation, Or, The Development of the Earth and Its Inhabitants by the Action of Natural Causes: A Popular Exposition of the Doctrine of Evolution in General, and of that of Darwin, Goethe, and Lamarck in Particular. From the 8^th^ German Ed. of Ernst Haeckel. (Vol. 1). K. Paul, Trench, Trübner & Company, Ltd

[B57] Halevy I., Alesker M., Schuster E.M., Popovitz-Biro R., and Feldman Y. (2017) A key role for green rust in the Precambrian oceans and the genesis of iron formations. Nat Geosci 10:135–139

[B191] Hand K.P., Carlson R.W., and Chyba C.F. (2007) Energy, chemical disequilibrium, and geological constraints on Europa. Astrobiology 7:1–18 18163875 10.1089/ast.2007.0156

[B58] Hansen H.C.B., Guldberg S., Erbs M., and Koch C.B. (2001) Kinetics of nitrate reduction by green rusts—effects of interlayer anion and Fe(II):Fe(III) ratio. Appl Clay Sci 18:81–91

[B59] Harold F.M. (2001) Gleanings of a chemiosmotic eye. BioEssays 23:848–855 11536297 10.1002/bies.1120

[B60] Haroon M.F., Hu S. S hi Y., Imelfort M., Keller J., Hugenholtz P., Yuan Z., and Tyson G.W. (2013) Anaerobic oxidation of methane coupled to nitrate reduction in a novel archaeal lineage. Nature 500:567–570 23892779 10.1038/nature12375

[B61] Heinen W. and Lauwers A.M. (1996) Organic sulfur compounds resulting from the interaction of iron sulfide, hydrogen sulfide and carbon dioxide in an anaerobic aqueous environment. Orig Life Evol Biosph 26:131–150 11536750 10.1007/BF01809852

[B62] Helz G. R., Erickson B.E., and Vorlicek T.P. (2014) Stabilities of thiomolybdate complexes of iron; implications for retention of essential trace elements (Fe, Cu, Mo) in sulfidic waters. Metallomics 6:1131–1140 24226648 10.1039/c3mt00217a

[B63] Herschy B., Whicher A., Camprubi E., Watson C., Dartnell L., Ward J., Evans J.R.G., and Lane N. (2014) An origin-of-life reactor to simulate alkaline hydrothermal vents. J Mol Evol 79:213–227 25428684 10.1007/s00239-014-9658-4 PMC4247476

[B64] Hirschmann M.M. and Withers A.C. (2008) Ventilation of CO_2_ from a reduced mantle and consequences for the early Martian greenhouse. Earth Planet Sci Lett 270:147–155

[B65] Hoffmann P.M. (2012) Life's Ratchet: How Molecular Machines Extract Order from Chaos, Basic Books, New York

[B66] Horita J. and Berndt M.E. (1999) Abiogenic methane formation and isotopic fractionation under hydrothermal conditions. Science 285:1055–1057 10446049 10.1126/science.285.5430.1055

[B67] Huber C. and Wächtershäuser G. (1997) Activated acetic acid by carbon fixation on (Fe,Ni)S under primordial conditions. Science 276:245–247 9092471 10.1126/science.276.5310.245

[B68] Hu R., Kass D.M., Ehlmann B.L., and Yung Y.L. (2015) Tracing the fate of carbon and the atmospheric evolution of Mars. Nat Commun 6:10003 26600077 10.1038/ncomms10003 PMC4673500

[B69] Itaya K., Chang H.C., and Uchida I. (1987) Anion-exchanged clay (hydrotalcite-like compounds) modified electrodes. Inorg Chem 26:624–626

[B70] Johannessen K.C., Vander Roost J., Dahle H., Dundas S.H., Pedersen R.B., and Thorseth I.H. (2016) Environmental controls on biomineralization and Fe-mound formation in a low-temperature hydrothermal system at the Jan Mayen Vent Fields. Geochim Cosmochim Acta 202:101–123

[B71] Johnson C.A., Murayama M., Küsel K., and Hochella M.F. (2015). Polycrystallinity of green rust minerals and their synthetic analogs: implications for particle formation and reactivity in complex systems. Am Miner 100:2091–2105

[B72] Kampschreur M.J., Kleerebezem R., de Vet W.W., and van Loosdrecht M.C. (2011) Reduced iron induced nitric oxide and nitrous oxide emission. Water Res 45:5945–5952 21940030 10.1016/j.watres.2011.08.056

[B73] Kaster A.-K., Moll J., Parey K., and Thauer R.K. (2011) Coupling of ferredoxin and heterodisulfide reduction via electron bifurcation in hydrogenotrophic methanogenic Archaea. Proc Natl Acad Sci USA 108:2981–2986 21262829 10.1073/pnas.1016761108 PMC3041090

[B74] Keene F.R. (1993) Thermodynamic, kinetic, and product considerations in carbon dioxide reactivity. In Electrochemical and Electrocatalytic Reactions of Carbon Dioxide, edited by Sullivan B.P., Krist K., and Guard H.E., Elsevier, Amsterdam, pp 118–144

[B75] Kelley D.S. and Früh-Green G.L. (1999) Abiogenic methane in deep-seated mid-ocean ridge environments: insights from stable isotope analyses. J Geophys Res 104:10439–10460

[B76] Kellosalo J., Kajander T., Kogan K., Pokharel K., and Goldman A. (2012) The structure and catalytic cycle of a sodium-pumping pyrophosphatase. Science 337:473–476 22837527 10.1126/science.1222505

[B77] Konn C., Charlou J.L., Holm N.G., and Mousis O. (2015) The production of methane, hydrogen, and organic compounds in ultramafic-hosted hydrothermal vents of the Mid-Atlantic Ridge. Astrobiology 15:81–399 10.1089/ast.2014.1198 PMC4442600 25984920

[B78] Koonin E.V. and Martin W. (2005) On the origin of genomes and cells within inorganic compartments. Trends Genet 21:647–654 16223546 10.1016/j.tig.2005.09.006 PMC7172762

[B79] Kovacs A.L. (1989) Degeneracy and asymmetry in biology. Nonlinear Structures in Physical Systems: Pattern Formation, Chaos, and Waves. Proceedings of the Second Woodward Conference San Jose State University 1117–18, 1989 Springer Science & Business Media, p 325

[B80] Lane N. (2010) Why are cells powered by proton gradients. Nat Ed 3:18

[B81] Lane N. and Martin W.F. (2012) The origin of membrane bioenergetics. Cell 151:1406–1416 23260134 10.1016/j.cell.2012.11.050

[B82] Lane N., Allen J.F., and Martin W. (2010) How did LUCA make a living? Chemiosmosis in the origin of life. Bioessays 32:271–280 20108228 10.1002/bies.200900131

[B83] Lang S.Q., Butterfield D.A., Schulte M., Kelley D.S., and Lilley M.D. (2010) Elevated concentrations of formate, acetate and dissolved organic carbon found at the Lost City hydrothermal field. Geochim Cosmochim Acta 74:941–952

[B84] Lang S.Q., Früh-Green G.L., Bernasconi S.M., Lilley M.D., Proskurowski G., Méhay S., and Butterfield D.A. (2012) Microbial utilization of abiogenic carbon and hydrogen in a serpentinite-hosted system. Geochim Cosmochim Acta 92:82–99

[B85] Lazar C., Cody G.D., and Davis J.M. (2015) A kinetic pressure effect on the experimental abiotic reduction of aqueous CO_2_ to methane from 1 to 3.5 kbar at 300° C. Geochim Cosmochim Acta 151:34–48

[B86] Lazar C., McCollom T.M., and Manning C.E. (2012) Abiogenic methanogenesis during experimental komatiite serpentinization: implications for the evolution of the early Precambrian atmosphere. Chem Geol 326:102–112

[B87] Leduc S. (1911) The Mechanism of Life, Rebman Ltd., London

[B88] Loeffler M.J. and Hudson R.L. (2015) Descent without modification? The thermal chemistry of H_2_O_2_ on Europa and other Icy Worlds. Astrobiology 15:453–461 26060983 10.1089/ast.2014.1195

[B89] Lollar B.S., Onstott T.C., Lacrampe-Couloume G., and Ballentine C.J. (2014) The contribution of the Precambrian continental lithosphere to global H_2_ production. Nature 516:379–382 25519136 10.1038/nature14017

[B90] Lyons J.R., Manning C., and Nimmo F. (2005) Formation of methane on Mars by fluid-rock interaction in the crust. Geophys Res Lett 32:L13201

[B91] Maden B.E.H. (2000) Tetrahydrofolate and tetrahydromethanopterin compared: functionally distinct carriers in C-1 metabolism. Biochem J 350:609–629 10970772 PMC1221290

[B92] Mancinelli R.L. and McKay C.P. (1988) The evolution of nitrogen cycling. Orig Life Evol Biosph 18:311–325 11538360 10.1007/BF01808213

[B93] Martin W. and Russell M.J. (2007). On the origin of biochemistry at an alkaline hydrothermal vent. Philos Trans R Soc Lond B Biol Sci 362:1887–1925 17255002 10.1098/rstb.2006.1881 PMC2442388

[B94] Mayhew L.E., Ellison E.T., McCollom T.M., Trainor T.P., and Templeton A.S. (2013) Hydrogen generation from low-temperature water-rock reactions. Nat Geosci 6:478–484

[B95] McCammon C. (1997) Perovskite as a possible sink for ferric iron in the lower mantle. Nature 387:694–696

[B96] McCollom T.M. (2013) Laboratory simulations of abiotic hydrocarbon formation in Earth's deep subsurface. Rev Miner Geochem 75:467–494

[B97] McCollom T.M. and Bach W. (2009) Thermodynamic constraints on hydrogen generation during serpentinization of ultramafic rocks. Geochim Cosmochim Acta 73:856–875

[B98] McCollom T.M. and Donaldson C. (2016) Generation of hydrogen and methane during experimental low-temperature reaction of ultramafic rocks with water. Astrobiology 16:389–406 27267306 10.1089/ast.2015.1382

[B99] McCollom T.M. and Seewald J.S. (2001) A reassessment of the potential for reduction of dissolved CO_2_ to hydrocarbons during serpentinization of olivine. Geochim Cosmochim Acta 65:3769–3778

[B100] McCollom T.M. and Seewald J.S. (2003) Experimental constraints on the hydrothermal reactivity of organic acids and acid anions: I. Formic acid and formate. Geochim Cosmochim Acta 67:3625–3644

[B101] McCollom T.M. and Seewald J.S. (2007) Abiotic synthesis of organic compounds in deep-sea hydrothermal environments. Chem Rev 107:382–401 17253758 10.1021/cr0503660

[B102] McCollom T.M. and Seewald J.S. (2013) Serpentinites, hydrogen, and life. Elements 9:129–134

[B103] McDermott J.M., Seewald J.S., German C.R., and Sylva S.P. (2015) Pathways for abiotic organic synthesis at submarine hydrothermal fields. Proc Natl Acad Sci 112:7668–7672 26056279 10.1073/pnas.1506295112 PMC4485091

[B104] McGlynn S.E. (2017) Energy metabolism during anaerobic methane oxidation in ANME archaea. Microbes Environ 32:5–13 28321009 10.1264/jsme2.ME16166 PMC5371075

[B105] McGlynn S.E., Mulder D.W., Shepard E.M., Broderick J.B., and Peters J.W. (2009) Hydrogenase cluster biosynthesis: organometallic chemistry nature's way. Dalton Trans 22:4274–4285 10.1039/b821432h 19662302

[B106] McSween H.Y. and Huss G.R. (2010) Cosmochemistry. Cambridge University Press, Cambridge

[B107] McSween H.Y., Taylor G.J., and Wyatt M.B. (2009) Elemental composition of the Martian crust. Science 324:736–739 19423810 10.1126/science.1165871

[B108] Mereschkowsky C. (1910) Theorie der zwei Plasmaarten als Grundlage der Symbiogenesis, einer neuen Lehre von der Entstehung der Organismen. Biol Centralbl 30:278–288; 289–303: 321–347; 353–367

[B109] Mielke R.E., Russell M.J., Wilson P.R., McGlynn S.E., Coleman M., Kidd R., and Kanik I. (2010) Design, fabrication, and test of a hydrothermal reactor for origin-of-life experiments. Astrobiology 10:799–810 21087160 10.1089/ast.2009.0456

[B110] Mitchell P. (1961) Coupling of phosphorylation to electron and hydrogen transfer by a chemiosmotic type of mechanism. Nature 191:144–148 13771349 10.1038/191144a0

[B111] Mitchell P. (1975) The protonmotive Q cycle: a general formulation. FEBS Lett 59:137–139 1227927 10.1016/0014-5793(75)80359-0

[B112] Mitchell P. (1976) Possible molecular mechanisms of the protonmotive function of cytochrome systems. J Theor Biol 62:27–367 10.1016/0022-5193(76)90124-7 186667

[B113] Moiseyev A.N. (1968) The Wilbur Springs quicksilver district (California) example of a study of hydrothermal processes by combining field geology and theoretical geochemistry. Econ Geol 63:169–181

[B114] Mousis O., Chassefière E., Holm N.G., Bouquet A., Waite J.H., Geppert W.D., Picaud S., Aikawa Y., Ali-Dib M., Charlou J.L., and Rousselot P. (2015) Methane clathrates in the solar system. Astrobiology 15:308–326 25774974 10.1089/ast.2014.1189

[B115] Neal C. and Stanger G. (1984) Calcium and magnesium hydroxide precipitation from alkaline groundwater in Oman, and their significance to the process of serpentinization. Min Mag 48:237–241

[B116] Nealson K.H. (1997). The limits of life on Earth and searching for life on Mars. J Geophys Res Planets 102:23675–23686 11541236

[B117] Neubeck A., Duc N.T., Bastviken D., Crill P., and Holm N.G. (2011) Formation of H_2_ and CH_4_ by weathering of olivine at temperatures between 30° and 70°C. Geochem Trans 12:6 21707970 10.1186/1467-4866-12-6 PMC3157414

[B118] Nitschke W. and Russell M.J. (2009) Hydrothermal focusing of chemical and chemiosmotic energy, supported by delivery of catalytic Fe, Ni, Mo/W, Co, S and Se, forced life to emerge. J Mol Evol 69:481–496 19911220 10.1007/s00239-009-9289-3

[B119] Nitschke W. and Russell M.J. (2011) Redox bifurcations; how they work and what they mean to extant life and (potentially) to its inorganic roots. BioEssays 34:106–10922045626

[B120] Nitschke W. and Russell M.J. (2013) Beating the acetyl co-enzyme-A pathway to the origin of life. Philos Trans R Soc Lond B Biol Sci 368, doi:10.1098/rstb.2012.0258 PMC3685460 23754811

[B121] Paukert A.N., Matter J.M., Kelemen P.B., Shock E.L., and Havig J.R. (2012) Reaction path modeling of enhanced in situ CO_2_ mineralization for carbon sequestration in the peridotite of the Samail Ophiolite, Sultanate of Oman. Chem Geol 330:86–100

[B122] Peters J.W., Miller A.F., Jones A.K., King P.W., and Adams M.W. (2016) Electron bifurcation. Curr Op Chem Biol 31:146–152 10.1016/j.cbpa.2016.03.007 27016613

[B123] Pfeffer W. (1877) Osmotische Untersuchungen; English translation, *Osmotic Investigations*, Van Nostrand Reinhold: New York, 1985

[B124] Pilet E., Nitschke W., Rappaport F., Soulimane T., Lambry J.C., Liebl U., and Vos M.H. (2004) NO binding and dynamics in reduced heme-copper oxidases *aa*_3_ from *Paracoccus denitrificans* and *ba*_3_ from *Thermus thermophilus*. Biochemistry 43:14118–14127 15518562 10.1021/bi0488808

[B125] Pineau F. and Mathez E.A. (1990) Carbon isotopes in xenoliths from the Hualalai Volcano, Hawaii, and the generation of isotopic variability. Geochim Cosmochim Acta 54:217–227

[B126] Pinske C. and Sargent F. (2016) Exploring the directionality of *Escherichia coli* formate hydrogenlyase: a membrane-bound enzyme capable of fixing carbon dioxide to organic acid. Microbiol Open 5:721–737 10.1002/mbo3.365 PMC5061711 27139710

[B127] Prigogine I. and Nicolis G. (1967) On symmetry-breaking instabilities in dissipative systems. J Chem Phys 46:3542–3550

[B128] Prigogine I. and Nicolis G. (1989) Exploring Complexity: An Introduction. Freeman and Co. Ltd., München

[B129] Proskurowski G., Lilley M.D., Seewald J.S., Früh-Green G.L., Olson E.J., Lupton J.E., Sylva S.P., and Kelley D.S. (2008) Abiogenic hydrocarbon production at Lost City hydrothermal field. Science 319:604–607 18239121 10.1126/science.1151194

[B130] Reeves E.P., McDermott J.M., and Seewald J.S. (2014) The origin of methanethiol in midocean ridge hydrothermal fluids. Proc Natl Acad Sci 111:5474–5479 24706901 10.1073/pnas.1400643111 PMC3992694

[B131] Roger M., Brown F., Gabrielli W., and Sargent F. (2017) Efficient hydrogen-dependent carbon dioxide reduction by *Escherichia coli*. bioRxiv, 169854 10.1016/j.cub.2017.11.050 PMC5772173 29290558

[B132] Roldan A., Hollingsworth N., Roffey A., Islam H.-U, Goodall J.B.M., Catlow C.R.A., Darr J.A., Bras W., Sankar G., Holt K.B., Hogarth G., and de Leeuw N.H. (2015) Bio-inspired CO_2_ conversion by iron sulfide catalysts under sustainable conditions. Chem Commun 51:7501–7504 10.1039/c5cc02078f 25835242

[B133] Ruby C., Abdelmoula M., Naille S., Renard A., Khare V., Ona-Nguema G., Morin G., and Génin J.-M.R. (2010) Oxidation modes and thermodynamics of FeII–III oxyhydroxycarbonate green rust: dissolution–precipitation versus in situ deprotonation. Geochim Cosmochim Acta 74:953–966

[B134] Russell M.J. and Beckett P. (2017) Is helicoidal green rust the missing link between hydrothermal chemistry and biochemistry? Astrobiology Science Conference 2017 (LPI Contrib. No. 1965), Tempe, Arizona 2017 https://www.hou.usra.edu/meetings/abscicon2017/pdf/3192.pdf

[B135] Russell M.J. and Hall A.J. (1997) The emergence of life from iron monosulphide bubbles at a submarine hydrothermal redox and pH front. J Geol Soc 154:377–402 10.1144/gsjgs.154.3.0377 11541234

[B136] Russell M.J. and Hall A.J. (2006) The onset and early evolution of life. In Evolution of Early Earth's Atmosphere, Hydrosphere, and Biosphere—Constraints from Ore Deposits, Geological Society of America Memoir 198, edited by Kesler S. and Ohmoto H., Geological Society of America, Boulder, CO, pp 1–32

[B137] Russell M.J. and Martin W. (2004) The rocky roots of the acetyl coenzyme-A pathway. Trends Biochem Sci 24:358–363 10.1016/j.tibs.2004.05.007 15236743

[B138] Russell M.J., Hall A.J., and Turner D. (1989). In vitro growth of iron sulphide chimneys: possible culture chambers for origin-of-life experiments. Terra Nova 1:238–241

[B139] Russell M.J., Daniel R.M., Hall A.J., and Sherringham J. (1994) A hydrothermally precipitated catalytic iron sulphide membrane as a first step toward life. J Mol Evol 39:231–243

[B140] Russell M.J., Hall A.J., and Mellersh A.R. (2003) On the dissipation of thermal and chemical energies on the early Earth: the onsets of hydrothermal convection, chemiosmosis, genetically regulated metabolism and oxygenic photosynthesis. In Natural and Laboratory-Simulated Thermal Geochemical Processes, edited by Ikan R., Kluwer Academic Publishers, Dordrecht, pp 325–388

[B141] Russell M.J., Hall A.J., Fallick A.E., and Boyce A.J. (2005) On hydrothermal convection systems and the emergence of life. Econ Geol 100:419–438

[B142] Russell M.J., Nitschke W., and Branscomb E. (2013) The inevitable journey to being. Philos Trans R Soc Lond B Biol Sci 368, doi:10.1098/rstb.2012.0254 PMC3685457 23754808

[B143] Russell M.J., Barge L.M., Bhartia R., Bocanegra D., Bracher P.J., Branscomb E., Kidd R., McGlynn S.E., Meier D.H., Nitschke W., Shibuya T., Vance S., White L., and Kanik I. (2014). The drive to life on wet and icy worlds. Astrobiology 14:308–343 24697642 10.1089/ast.2013.1110 PMC3995032

[B144] Russell M.J., Murray A.E., and Hand K.P. (2017) The possible emergence of life and differentiation of a shallow biosphere on irradiated icy worlds: the example of Europa. Astrobiology 17; doi: 10.1089/ast.2016.1600 PMC5729856 29016193

[B145] Scheller S., Yu H., Chadwick G.L., McGlynn S.E., and Orphan V.J. (2016). Artificial electron acceptors decouple archaeal methane oxidation from sulfate reduction. Science 351:703–707 26912857 10.1126/science.aad7154

[B146] Schoepp-Cothenet B., van Lis R., Atteia A., Baymann F., Capowiez L., Ducluzeau A.L., Duval S., ten Brink F., Russell M.J., and Nitschke W. (2013) On the universal core of bioenergetics. Biochim Biophys Acta 1827:79–93 22982447 10.1016/j.bbabio.2012.09.005

[B147] Schoonen M.A.A., Xu Y., and Bebie J. (1999) Energetics and kinetics of the prebiotic synthesis of simple organic acids and amino acids with the FeS-H_2_S/FeS_2_ redox couple as reductant. Orig Life Evol Biosph 29:5–32 10077866 10.1023/a:1006558802113

[B148] Schrödinger E. (1944) What is life? The physical aspects of the living cell. Cambridge, UK: Cambridge, University Press

[B149] Schuchmann K. and Muller V. (2013) Direct and reversible hydrogenation of CO_2_ to formate by a bacterial carbon dioxide reductase. Science 342:1382–1385 24337298 10.1126/science.1244758

[B150] Seewald J.S., Zolotov M.Y., and McCollom T. (2006) Experimental investigation of single carbon compounds under hydrothermal conditions. Geochim Cosmochim Acta 70:446–460

[B151] Seyfried W.E., Pester N.J., Tutolo B.M., Ding K. (2015) The Lost City hydrothermal system: constraints imposed by vent fluid chemistry and reaction path models on subseafloor heat and mass transfer processes. Geochim Cosmochim Acta 163:59–79

[B152] Shan J., Huang W., Nguyen L., Yu Y., Zhang S., Li Y., Frenkel A.I., and Tao F. (2014) Conversion of methane to methanol with a bent mono(μ-oxo)dinickel anchored on the internal surfaces of micropores. Langmuir 30:8558–8569 24896721 10.1021/la501184b

[B153] Shibuya T., Russell M.J., and Takai K. (2016) Free energy distribution and hydrothermal mineral precipitation in Hadean submarine alkaline vent systems; Importance of iron redox reactions under anoxic conditions. Geochim Cosmochim Acta 175:1–19

[B154] Shock E.L. (1992) Chemical environments of submarine hydrothermal systems. Orig Life Evol Biosph 22:67–107 11537554 10.1007/BF01808019

[B155] Shock E.L. and Boyd E.S. (2015) Principles of geobiochemistry. Elements 11:395–401

[B156] Shock E. and Canovas P. (2010) The potential for abiotic organic synthesis and biosynthesis at seafloor hydrothermal systems. Geofluids 10:161–192

[B157] Sojo V., Herschy B., Whicher A., Camprubí E., and Lane N. (2015) The origin of life in alkaline hydrothermal vents. Astrobiology 16:181–197 10.1089/ast.2015.1406 26841066

[B158] Sousa F. and Martin W.F. (2014) Biochemical fossils of the ancient transition from geoenergetics to bioenergetics in prokaryotic one carbon compound metabolism. Biochim Biophys Acta 1837:964–981 24513196 10.1016/j.bbabio.2014.02.001

[B159] Starokon E.V., Parfenov M.V., Arzumanov S.S., Pirutko L.V., Stepanov A.G., and Panov G.I. (2013) Oxidation of methane to methanol on the surface of FeZSM-5 zeolite. J Catal 300:47–54

[B160] Stern J.C., Sutter B., McKay C.P., Navarro-Gonzalez R., Freissinet C., Conrad P.G., Mahaffy P.R., Archer P.D., Ming D.W., Niles P.B., Zorzano M.-P., Martin-Torres F.J., and the MSL Science Team. (2015) The nitrate/perchlorate ratio on Mars as an indicator for habitability. In Lunar Planet Sci Conf 46:2590

[B161] Strauss H. (1989) Carbon and sulfur isotope data for carbonaceous metasediments from the Kidd Creek massive sulfide deposit and vicinity, Timmins, Ontario. Econ Geol 84:959–962

[B162] Suda K., Ueno Y., Yoshizaki M., Nakamura H., Kurokawa K., Nishiyama E., Yoshino K., Hongoh Y., and Kawachi K. (2014) Origin of methane in serpentinite-hosted hydrothermal systems: the CH_4_–H_2_–H_2_O hydrogen isotope systematics of the Hakuba Happo hot spring. Earth Planet Sci Lett 386:112–125

[B163] Thauer R.K., Kaster A.K., Seedorf H., Buckel W., and Hedderich R. (2008) Methanogenic Archaea: ecologically relevant differences in energy conservation. Nat Rev Microbiol 6:579–591 18587410 10.1038/nrmicro1931

[B164] Tosca N.J., Guggenheim S., and Pufahl P.K. (2016) An authigenic origin for Precambrian greenalite: Implications for iron formation and the chemistry of ancient seawater. Geol Soc Am Bull 128:511–530

[B165] Traube M. (1867) Experimente zur Theorie der Zellenbildung und Endosmose. Arch Anat Physiol Wiss Med 87:129

[B166] Trolard F. and Bourrié G. (2012) Fougerite a natural layered double hydroxide in gley soil: habitus, structure, and some properties. In Clay Minerals in Nature: Their Characterization, Modification and Application, edited by Valaskova M. and Martynkova G.S., InTech, Rijeka, Croatia, pp 171–188

[B167] Tsai J.Y., Kellosalo J., Sun Y.J., and Goldman A. (2014) Proton/sodium pumping pyrophosphatases: the last of the primary ion pumps. Curr Op Struct Biol 27:38–47 10.1016/j.sbi.2014.03.007 24768824

[B168] Wadhwa M. (2001) Redox state of Mars' upper mantle and crust from Eu anomalies in shergottite pyroxenes. Science 291:1527–1530 11222854 10.1126/science.1057594

[B169] Wadhwa M. (2008) Redox conditions on small bodies, the Moon and Mars. In Oxygen in the Solar System, edited by MacPherson G.J., Mittlefehldt D.W., Jones J.H., and Simon S.B., Rev Mineral Geochem 68:493–510

[B170] Waite J.H. Jr, Lewis W.S., Magee B.A., Lunine J.I., McKinnon W.B., Glein C.R., Mousis O., Young D.T., Brockwell T., Westlake J. and Nguyen M.J. (2009) Liquid water on Enceladus from observations of ammonia and ^40^Ar in the plume. Nature 460:487–490

[B171] Wander M.C., Rosso K.M., and Schoonen M.A. (2007) Structure and charge hopping dynamics in green rust. J Phys Chem C 111:11414–11423

[B172] Wang W. and Gong J. (2011) Methanation of carbon dioxide: an overview. Front Chem Sci Eng 5:2–10

[B173] Wang W., Song Y., Wang X., Yang Y., and Liu X. (2015) Alpha-oxo acids assisted transformation of FeS to Fe_3_S_4_ at low temperature: implications for abiotic, biotic, and prebiotic mineralization. Astrobiology 15:1043–1051 26625153 10.1089/ast.2015.1373

[B174] Watanabe S., Mishima K., and Matsuo S. (1983) Isotopic ratios of carbonaceous materials incorporated in olivine crystals from the Hualalai Volcano, Hawaii. An approach to mantle carbon. Geochem J 17:95–104

[B175] Weiss M.C., Sousa F.L., Mrnjavac N., Neukirchen S., Roettger M., Nelson-Sathi S., and Martin W.F. (2016) The physiology and habitat of the last universal common ancestor. Nat Microbiol 1:16116 27562259 10.1038/nmicrobiol.2016.116

[B176] White L.M. (2013) Exploring inorganic catalytic pathways for CO_2_ reduction: metal-oxide nanowires and iron sulfide minerals. PhD Thesis, University of California, Santa Barbara

[B177] White L.M., Bhartia R., Stucky G.D., Kanik I., and Russell M.J. (2015) Mackinawite and greigite in ancient alkaline hydrothermal chimneys: identifying potential key catalysts for emergent life. Earth Planet Sci Lett 430:105–114

[B178] Wikström M. K. and Berden J. A. (1972) Oxidoreduction of cytochrome b in the presence of antimycin. Biochim Biophys Acta 283:403–420 4346389 10.1016/0005-2728(72)90258-7

[B179] Wilkin R.T. and Beak D.G. (2017) Uptake of nickel by synthetic mackinawite. Chem Geol 462:15–29 10.1016/j.chemgeo.2017.04.023 PMC6145480 30245527

[B180] Windman T., Zolotova N., Schwandner F., and Shock E. L. (2007) Formate as an energy source for microbial metabolism in chemosynthetic zones of hydrothermal ecosystems. Astrobiology 7:873–890 18163868 10.1089/ast.2007.0127

[B181] Woese C.R., Kandler O., and Wheelis M.L. (1990) Towards a natural system of organisms: proposal for the domains Archaea, Bacteria and Eukarya. Proc Natl Acad Sci USA 87:4576–4579 2112744 10.1073/pnas.87.12.4576 PMC54159

[B182] Wolfe R. (2014) Early days with Carl. RNA Biol 11:175 24572323 10.4161/rna.27429 PMC4008545

[B183] Wong M.L., Charnay B.D., Gao P., Yung Y.L., and Russell M.J. (2017a) Nitrogen oxides in early Earth's atmosphere as electron acceptors for life's emergence. Astrobiology 17:000–000 10.1089/ast.2016.1473 29023147

[B184] Wong M.L., Gao P., Friedson A.J., Yung Y.L., and Russell M.J. (2017b) A methane-rich early Mars: implications for habitability and the emergence of life. Astrobiology Science Conference 2017 (LPI Contrib. No. 1965) Tempe, Arizona 2017 https://www.hou.usra.edu/meetings/abscicon2017/pdf/3524.pdf

[B185] Wood B.J. (2000) Phase transformations and partitioning relations in peridotite under lower mantle conditions. Earth Planet Sci Lett 174:341–354

[B186] Wood B.J., Walter M.J., and Wade J. (2006) Accretion of the Earth and segregation of its core. Nature 441:825–833 16778882 10.1038/nature04763

[B187] Wu D., Shao B., Fu M., Luo C., and Liu Z. (2015) Denitrification of nitrite by ferrous hydroxy complex: effects on nitrous oxide and ammonium formation. Chem Eng J 279:149–155

[B188] Wu J.F., Yu S.M., Wang W.D., Fan Y.X., Bai S., Zhang C.W., Gao Q., Huang J., and Wang W. (2013) Mechanistic insight into the formation of acetic acid from the direct conversion of methane and carbon dioxide on zinc-modified H–ZSM-5 zeolite. JACS 135:13567–13573 10.1021/ja406978q 23981101

[B189] Yamaguchi A., Yamamoto M., Takai K., Ishii T., Hashimoto K., and Nakamura R. (2014) Electrochemical CO_2_ reduction by Ni-containing iron sulfides: how is CO_2_ electrochemically reduced at bisulfide-bearing deep-sea hydrothermal precipitates? Electrochim Acta 141:311–318

[B190] Zolotov M.Y. and Kargel J.S. (2009) On the chemical composition of Europa's icy shell, ocean, and underlying rocks. In Europa, edited by Pappalardo R.T., McKinnon W.B., Khurana K., University of Arizona Press, Tucson, AZ, pp 431–457

